# Dual head screw hip nailing for trochanteric fractures

**DOI:** 10.1051/sicotj/2017049

**Published:** 2017-10-18

**Authors:** Andreas F. Mavrogenis, Vasilios G. Igoumenou, Panayiotis D. Megaloikonomos, George N. Panagopoulos, Ioannis P. Galanopoulos, Christos Th. Vottis, Eirinaios Karamanis, Panayiotis Koulouvaris, Panayiotis J. Papagelopoulos

**Affiliations:** 1 First Department of Orthopaedics, National and Kapodistrian University of Athens, School of Medicine 41 Ventouri Str. 15562 Athens Greece

**Keywords:** Hip fractures, Trochanteric, Hip nailing, Dual head screws

## Abstract

*Introduction*: There are limited information and inconclusive results for dual head screw intramedullary hip nails for trochanteric fractures. Therefore, we performed a prospective study to evaluate the healing of fractures, and survival, function, and complications of patients operated with this implant.

*Methods*: We prospectively studied 79 patients (61 women and 18 men; mean age: 84.7 years; range: 65–96 years) with a low-energy trochanteric fracture, treated with a dual head screw intramedullary hip nail from 2013 to 2016. The mean follow-up was 2.1 years (range: 1–3 years); seven patients were lost to follow up. This left 72 patients for further analysis. We evaluated the healing of fractures, and survival, function, and complications of patients.

*Results*: Fracture healing was evident in 70 patients (97.2%) at 2–3 months postoperatively. One patient experienced cut-out and z-effect phenomenon of the head screws. Another patient experienced a periprosthetic femoral diaphysis fracture at the distal tip of the nail. A third patient experienced an acute postoperative superficial skin infection that was treated successfully with wound dressing changes and a course of antibiotics. Sixteen patients (22.2%) deceased within 12 months postoperatively. In the remaining patients, the Harris Hip Score (HHS) at 12 months postoperatively was excellent in 16 (28.6%), good in 23 (41.1%), fair in 10 (17.8%), and poor in 7 patients (12.5%). The function declined after the patients’ fracture. Fair and poor results were related to age > 85 years, poor pre-fracture level of function, and AO/OTA-31-A3 fracture types.

*Conclusion*: The dual head screw intramedullary hip nail is associated with high healing and low complication rates for intertrochanteric fractures. The function of the patients is good or excellent in most cases; however, it declines, especially for those patients with age > 85 years, poor pre-fracture level of function, and AO/OTA-31-A3 fracture types.

## Introduction

Intertrochanteric (extracapsular) fractures represent a large subgroup of hip fractures accounting for 33–50% of all hip fractures [1–3]. The majority of fractures occur in elderly patients [1]. Their mortality rate reaches 10% at hospital admission and 30% at 12 months postoperatively. Approximately 50% of the elderly patients who survive their hip fracture are expected to recover their pre-fracture functional level, while 25% of those who were independent before their fracture will require admission to a home for the elderly [2, 4].

The treatment of intertrochanteric fractures has evolved along with advances in the design of the implants used for osteosynthesis; however, there remains conflicting evidence to guide the choice of implant [3, 5, 6]. Intramedullary nails for hip fracture osteosynthesis met a striking increase from 3% in 1999 to 67% in 2006. This change has been noted, despite a lack of evidence in the literature and potentially known complications [5–7]. Additionally, there is no conclusive evidence on the specific characteristics of the intramedullary nails for extracapsular hip fractures [3, 5–11]. Therefore, to enhance the literature, we performed this study to evaluate the union of the trochanteric fractures, and survival, function, and complications of the patients operated with an intramedullary dual lag screw hip nail.

## Materials and methods

We prospectively studied 79 elderly patients with a low-energy AO/OTA 31-A extra-articular fracture of the trochanteric area [12], admitted and treated at our Institution with the Veronail^®^ (Orthofix Srl, Bussolengo, Verona, Italy) dual head screw intramedullary hip nail from January 2013 to January 2016. There were 61 women and 18 men with a mean age of 84.7 years (range: 65–96 years). Forty-five patients experienced an AO/OTA-31-A1 fracture, 20 patients experienced an AO/OTA-31-A2 fracture, and 14 patients experienced an AO/OTA-31-A3 fracture. Patients younger than 65 years of age, polytrauma patients, tumor patients with a pathologic hip fracture, and patients with previous ipsilateral hip or femur surgery possibly affecting functional outcome were excluded. The mean follow-up was 2.1 years (range: 1–3 years); seven patients were lost to follow-up. This left 72 patients for further analysis; there were 39 patients with an AO/OTA-31-A1 fracture, 19 patients with an AO/OTA-31-A2 fracture, and 14 patients with an AO/OTA-31-A3 fracture. All patients or their relatives gave written informed consent for their data to be included in this study. This study was approved by the Institutional Review Board/Ethics Committee of the authors’ institution.

The procedures were performed on a fracture table under spinal anesthesia. Fluoroscopy-guided closed reduction of the fracture was achieved. Through the lateral approach, the trochanteric entry point was identified and after proximal reaming, the Veronail^®^ (Orthofix Srl, Bussolengo, Verona, Italy) intramedullary hip nail was gently advanced. The optimal position of the nail was evaluated by the level of the distal cephalic lag screw; this should be distal to the midaxis of the femoral neck, close to or even onto the medial cortex so that the proximal screw is placed as close as possible to the center of the head in anteroposterior images. In all cases, static distal interlocking fixation was done.

The Veronail^®^ (Orthofix Srl, Bussolengo, Verona, Italy) intramedullary hip nail is a 200 mm long intramedullary nail with a proximal diameter of 15 mm and a distal diameter of 10 mm. The nail incorporates two head screws that can be inserted in two fixation configurations: two parallel cephalic sliding screws or two convergent fixed screws. The parallel sliding screws allow 10–40 mm of sliding (depending on the length of the screw). The converging screws are locked into the nail and provide secure locked fixation of the femoral neck and head. The choice of the proximal configuration depends on the clinical and biomechanical characteristics of the fracture. In the parallel configuration, the screw-nail angle is 128°; this configuration favors sliding and rotational stability for controlled fracture impaction. In the convergent configuration, the distal head screw has a 128° screw-nail angle and the proximal head screw a 120° angle; this configuration allows the head screws to be fitted in very narrow necks and provides stable fixation with locked screws. Trochanteric simple hip fractures can be treated with sliding head screws, while trochanteric multifragmentary hip fractures with an inherent risk of collapse are best treated with convergent screws. A convergent configuration may also be used if the femoral neck is too narrow to accommodate two parallel screws, and for subtrochanteric fractures. Distal locking can be either static or dynamic at the discretion of the treating surgeon based on the bone quality and fracture type [7].

Forty-six patients were treated within 48 h from their admission; the remaining were patients with severe comorbidities and/or under anticoagulation treatment for cardiovascular disorders. The mean duration of surgical operations was 42.3 min (range: 31–56 min), the mean fluoroscopy time was 56.9 s (range: 52–67 s), and the mean number of blood transfusion units the patients required during the hospital stay was 1.9 units (range: 0–4 units).

Postoperative rehabilitation included passive and active-assisted lower limb exercises, and mobilization in sitting position at day one. The patients were encouraged to mobilize with a walking frame and bear weight as tolerated depending on their general health status and compliance at day two. The patients were discharged from the hospital at a mean stay of five days (range: 3–12 days) with instructions for partial weight bearing with a walking frame for one month. No patient was discharged to his home living independently; 69 of the 79 patients (87.3%) were discharged to their home living with their relatives, and 10 of the 79 patients (11.4%) were discharged to a rehabilitation center (eight patients) or an elderly nursing facility (two patients). A routine postoperative follow-up examination was done at one, two, three, six, and 12 months, and then annually. For each postoperative year after the first, the patients or their relatives were contacted by a telephone call, were inquired for clinical details (to evaluate for survival and function), and were asked to send a radiograph of their hip (to evaluate for implant-related complications).

At baseline (preoperatively), we evaluated the age, AO/OTA type of fracture [12], and pre-fracture level of function (ability for independent ambulation, basic activities of daily living, and living independently). At immediate postoperative anteroposterior and lateral radiographs, we evaluated the quality of reduction in the amount of displacement and neck-shaft alignment (classified as good, acceptable, or poor) [13]. A good reduction had normal or slightly valgus neck-shaft alignment on the anteroposterior radiograph, less than 20° of angulation on the lateral, and displacement of less than 4 mm on either view. Acceptable reductions met the requirements as regards alignment or displacement, but not both. Poor reductions met neither criteria [13]. At follow-ups, we evaluated the patients’ survival and function with the Harris Hip Score (HHS) questionnaire [14], and fracture healing, tip-apex distance (TAD), caput-collum-diaphyseal (CCD) angle, leg length discrepancy (LLD) [15], and implant-related complications with radiographs of the hip. Fracture healing was evaluated by trabeculation across the fracture and obliteration of the fracture line. We used the TAD as a method of evaluating the head screw position of the implants. TAD is the sum of the distance from the tip of the lag screw to the apex of the femoral head on an anteroposterior radiograph and this distance on a lateral radiograph, after controlling for magnification. As a point of measurement of TAD for the dual head screw intramedullary nails used in this study, we used the tip of the proximal screw. The CCD angle is the angle formed between the longitudinal axes of the femoral neck and shaft. It normally measures approximately 126° in adults (coxa norma); an abnormally small angle is known as coxa vara and an abnormally large angle as coxa valga. The CCD of the fractured hip was compared to the contralateral. The LLD was evaluated in anteroposterior radiographs of the pelvis by measuring the vertical distance between the horizontal line drawn across the inferior aspect of the ischial tuberosities (pelvic reference) to the most prominent medial point on the lesser trochanters (femoral reference), corrected for magnification (true leg length discrepancy) [15].

Statistical analysis was done with the dependent *t*-test for paired samples. Data were tabulated in a Microsoft Excel^®^ sheet (Microsoft Corporation, Redmond, Washington, USA) and analyzed using the SPSS v. 18.0.0 (SPSS Inc., Chicago, IL, USA) statistical package for personal computers.

## Results

**Figure 1.**
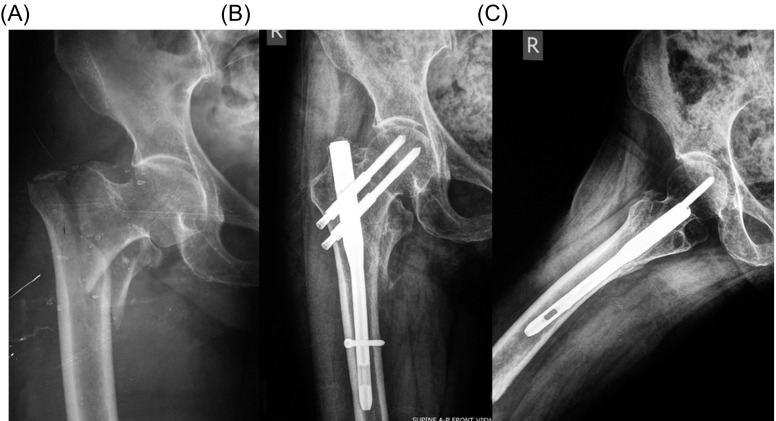
(A) Anteroposterior radiograph of the right hip of a 65-year-old woman shows an AO/OTA-31-A2 fracture. (B) Anteroposterior and (C) lateral radiographs of the right hip one year after surgical treatment show fracture healing. The patient was alive with excellent function.

**Figure 2.**
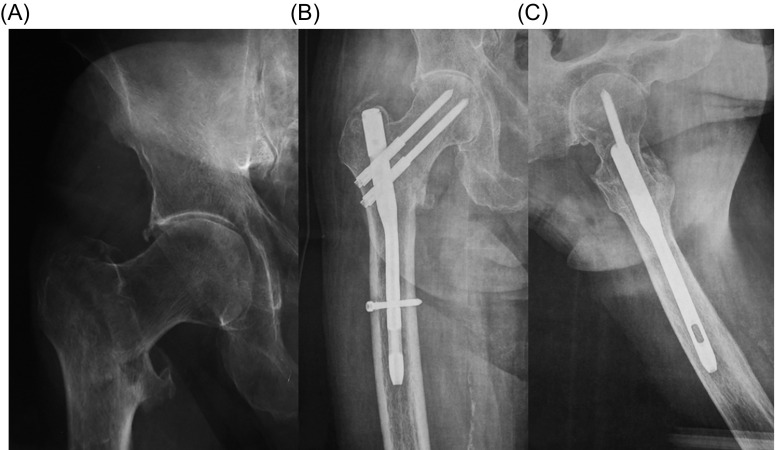
(A) Anteroposterior radiograph of the right hip of a 75-year-old man shows an AO/OTA-31-A1 fracture. (B) Anteroposterior and (C) lateral radiographs of the right hip nine months after surgical treatment show fracture healing. The patient was alive with excellent function.

**Figure 3.**
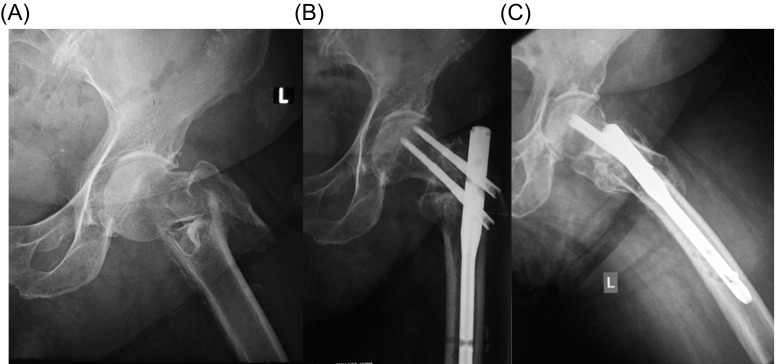
(A) Anteroposterior radiograph of the left hip of an 81-year-old woman shows an AO/OTA-31-A3 fracture. (B) Anteroposterior and (C) lateral radiographs of the left hip one year after surgical treatment show fracture healing. The patient was alive with good function.

The quality of reduction in immediate postoperative radiographs was good in 43 patients (59.7%), acceptable in 22 patients (30.6%), and poor in seven patients (9.7%). Fracture healing was evident in 70 patients (97.2%) at 2–3 months postoperative radiographs (Figures 1–3). The mean TAD was 21.1 mm (range: 9–44 mm); the mean CCD was 127° (range: 125–130°) that is similar to the normal value of CCD for adults (126°) and the screw-nail angle of the implant used in the present series (128°), without any statistically significant difference with the contralateral hip (*p* < 0.001). The leg length discrepancy was less than 1 cm in 70 patients and 1–1.5 cm in two patients.

**Figure 4.**
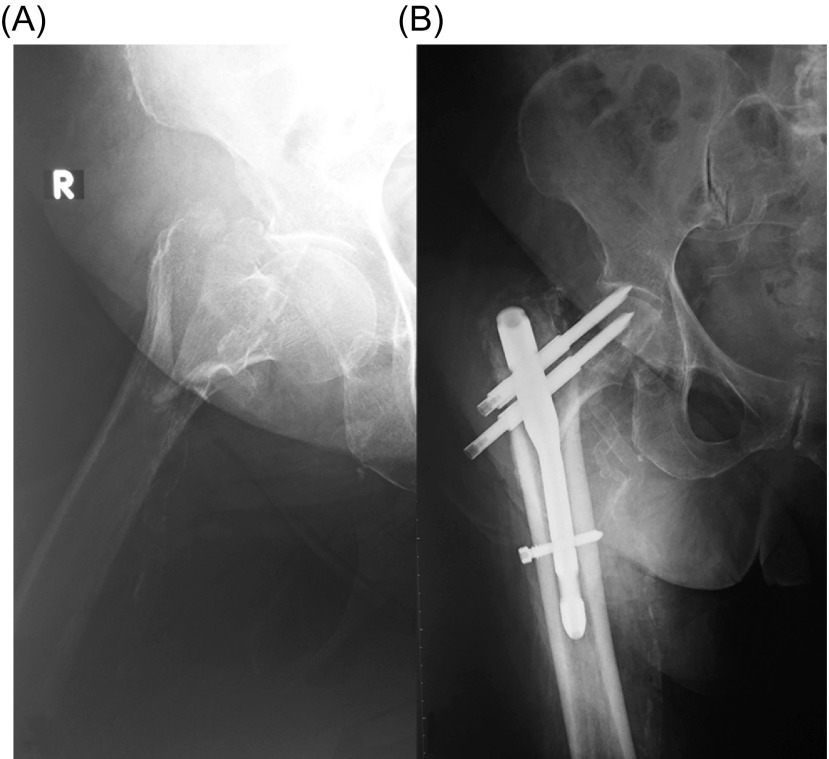
(A) Anteroposterior radiograph of the right hip of an 85-year-old woman shows an AO/OTA-31-A2 fracture. (B) Anteroposterior radiograph of the right hip eight weeks after surgical treatment shows screw cut-out and z-effect. The patient was alive with poor function and did not consent to further treatment.

One patient (1.4%) experienced cut-out and z-effect phenomenon of the cephalic screws two months postoperatively (Figure 4). In this patient, the head screws were initially positioned improperly (TAD, 44 mm) because of poorly closed reduction of the fracture. The patient was offered a revision surgery, however, she did not consent to any further operation. Another patient (1.4%) experienced a periprosthetic femoral diaphysis fracture at the distal tip of the nail, after falling from standing position, six months postoperatively. In this patient, a revision surgery was done with closed reduction of the fracture and exchange of the short with a similar dual head screw long intramedullary nail. A third patient (1.4%) experienced an acute postoperative superficial infection that was treated successfully with wound dressing changes and a two-week course of antibiotics.

Sixteen patients (22.2%) deceased within 12 months of their fracture; these patients were not included in the clinical analysis of function at 12-month follow-up. This left 56 patients who were alive at 12-month follow-up; in this group of patients, analysis of function was done at 3, 6, and 12 months postoperatively. The HHS of the alive patients at three, six, and 12 months postoperatively was excellent in 14, 15, and 16 patients (25%, 26.8%, and 28.6%), good in 17, 19, and 23 patients (30.3%, 33.9%, and 41.1%), fair in 16, 14, and 10 patients (28.6%, 25%, and 17.8%), and poor in nine, eight, and seven patients (16.1%, 14.3%, and 12.5%), respectively. Fair and poor results were significantly related to age > 85 years, poor pre-fracture level of function (difficult ambulation), and AO/OTA-31-A3 fracture types (*p* < 0.001). The function declined after the patients’ fracture. Before their fracture, 38 patients (68%) were able for independent ambulation without aid (a cane or a frame), 46 patients (82.1%) were able for independent basic activities of daily living, and 40 patients (71.4%) were living independently, alone (19 patients), or with their relatives (21 patients). One year after their fracture, 14 patients (25%) were able for independent ambulation without aid, 28 patients (50%) were able for independent basic activities of daily living, and 10 patients (18%) were living independently (Table 1).

**Table 1.** A summary of the results of the patients included in this series.

Variables	Outcome/Measurements
Quality of reduction	Good (43 patients, 59.7%), acceptable (22 patients, 30.6%), poor (7 patients, 9.7%)
Fracture healing	97.2% (70 patients) at 2–3 months postoperatively
TAD	Mean: 21.1 mm; range: 9–44 mm
CCD	Mean: 127°; range: 125–130°
LLD	< 1 cm in 70 patients; 1–1.5 cm in two patients
Complications	Cut-out and z-effect phenomenon (one patient, 1.4%); periprosthetic femoral diaphysis fracture at the distal tip of the nail (one patient, 1.4%); acute postoperative superficial infection (one patient, 1.4%)
Survival*	78.8% (56 patients)
Function (HHS)*	Excellent (16 patients, 28.6%), good (23 patients, 41.1%), fair (10 patients, 17.8%), poor (seven patients, 12.5%)
Independent ambulation without aid*	14 patients (25%)
Independent basic activities of daily living*	28 patients (50%)
Independent living*	10 patients (18%)

* At 12 months follow-up, in 56 patients; HHS: Harris Hip Score; TAD: tip-apex distance; CCD: caput-collum-diaphyseal angle; LLD: leg length discrepancy.

## Discussion

Intramedullary hips nails are currently considered the implants of choice for the surgical treatment of patients with trochanteric fractures because of their biomechanical advantages, minimally invasive insertion technique, and limited soft tissue injury [3–11, 16–27]. In this article, we evaluated the outcome of the patients, and the fracture healing and related complications of a dual head screw intramedullary nail for trochanteric fractures. Our results showed a 22.2% incidence of death within 12 months after the hip fracture, a 69.7% rate of excellent and good function, a 97.2% rate of fracture healing, and a 4.2% rate of complications. The function declined after the patients’ fracture. Fracture healing was observed by 8–12 weeks postoperatively as confirmed clinically by the patients’ lack of pain and ability to ambulate independently with a cane or a frame, and radiographically. Unless bone loss or severe comminution is present, by 4–8 weeks after osteosynthesis of an intertrochanteric fracture, callus is beginning to bridge the fracture fragments in the femoral region and endosteal healing is bridging the metaphyseal region. At this time, the fracture is considered stable and no precautions are recommended if the patients are asymptomatic [28].

The plate and sliding screw extramedullary fixation has been the gold standard for the treatment of intertrochanteric fractures [22]. However, fracture collapse, medialization of the femur, and limb shortening were known complications related to this type of osteosynthesis [22]. Compared to plate and screw constructs, intramedullary hips nails are considered biomechanically superior for load transfer. Additionally, as minimally invasive techniques they are associated with a biological advantage that relates to shorter healing and recovery times and improved function for the patients [11, 17–27]. Probably, a reason why some surgeons still prefer the plate and sliding screw constructs for intertrochanteric fractures is the lower cost of these implants [18, 19]. A recent financial decision analysis [19] showed that plate and screw constructs are most cost-effective options for AO/OTA-31-A1 fractures, while for AO/OTA-31-A3 fractures, hip nails dominate due to the revision costs if a plate and screw construct was used. For AO/OTA-31-A2 fractures, a plate and screw construct is probably more cost-effective (70% of cases).

The hip nail used in this study is a dual head screw implant, with a sliding or locked configuration of the head screws depending on fracture comminution and subtrochanteric extension [7]. Dual head screw constructs are believed to improve rotational stability and bony purchase within the femoral head, and to resist cut-out and subsequent fixation failure [17, 21–23]. Based on biomechanical studies, dual head screw hip nails have shown significantly stronger fixation compared to single head screw hip nails when loaded to failure in an unstable intertrochanteric fracture model [21], and significantly stronger fixation and less rotational instability compared to plate and screw constructs when loaded with multidirectional dynamic forces [22]. Additionally, a dual head screw hip nail may be useful in patients with a small proximal femur because of the smaller diameter of the head screws [24].

Previous studies reported favorable outcomes for the patients and low complication rates of hip nails [7, 10, 25]. Unfortunately, function and independent living of the elderly patients with hip fractures will decline, and a percentage of them will die within 12 months after their fracture [4, 16]. In the present series, 16 patients (22.2%) deceased within 12 months of their fracture. By direct comparison of our data, fewer patients deceased if they were operated within 48 h after their fracture (seven patients) compared to those who were operated after 48 h (nine patients). However, this finding should be interpreted with caution because of the small number of patients and the potential bias by the patients’ comorbidities and overall clinical status. The alive patients had worse function compared to their pre-fracture level of function with respect to independent ambulation (25%), basic activities of living (50%), and independent living (18%). Fracture healing was observed in most patients by 8–12 weeks postoperatively. By that time, the fracture is considered stable; the patients were asymptomatic and were able to walk independently with a cane or a walking frame [28].

Complications of intramedullary nailing for hip fractures include risk for iatrogenic fracture or fracture comminution during nail insertion, suboptimal closed fracture reduction [17], cut-out of the head screw (range: 0–16%), femoral periprosthetic fracture (range: 0–5%) [18], nonunion (1%), infection (< 1%) [11], z-effect, and reverse z-effect phenomena that are unique complications of nails with dual head screws (range: 0–13.3%) [18, 20, 24, 29–32]. The z-effect involves lateral migration of the inferior head screw and medial migration of the superior screw; reverse z-effect is the opposite [29]. Other complications of intramedullary hip nailing include malalignment, false drilling, wrong lag screw length, and drill bit breakage during the interlocking procedure, external or internal malrotation (≥ 20°) of the femoral diaphysis, lengthening or shortening of the limb (≥ 2 cm), impaired bone healing, fracture collapse, implant failure, lag screw intrapelvic migration, neurovascular injury, secondary varus deviation, complications after implant removal, trochanteric pain, and refracture [30]. There are several factors that predispose to complications of intramedullary nailing for hip fractures including advanced age (poor bone quality), complex fracture patterns, fracture malreduction, and eccentric lag screw placement [11]. Fracture malreduction has been associated with three times more risk of failure [11]. Cut-out of the head screw is a consequence of incorrect placement of the screw, rather than the type of implant itself. The TAD is a useful index to assess the risk of head screw cutout. A TAD > 25 mm is associated with an increased risk of cut-out. The optimal head screw positioning is in the center of the neck and within 5 mm of subchondral bone of the femoral head [11]. Risk factors for z-effect phenomenon include improper entry point, severe osteoporosis, and medial cortex comminution [30]. To prevent z-effect in dual head screw implants, it is important to insert the distal head screw as close as possible to the calcar femoralis in order to achieve a better anchorage of the screw to dense bone and to achieve a position of the proximal head screw close to the center of the femoral neck [24]. Some surgeons [33] reported that in dual head screw hip nails, the insertion of a longer distal head screw is associated with less stress within the bone, the nail, and the proximal head screw, and less lateral sliding of the screw resulting in z-effect [33]. In the present study, cut-out and z-effect occurred in one patient with a TAD of 44 mm resulting from poor reduction and an improper head screw insertion technique. This rate is significantly lower compared to other dual head screw implants. This should be attributed to the design of the Veronail^®^ intramedullary hip nail that incorporates locking of the head screws to the nail. This design prevents the distal head screw from excessive varus force and cyclic loading that may cause the screw to toggle and back out, the head/neck segment to collapse into varus, and the proximal screw to migrate through the femoral head (z-effect) [34]. Several studies have also shown superiority of dual compared to single head screw hip nails with respect to periprosthetic femoral fractures [26, 27, 35]; this has been explained by the fact that a dual head screw construct predetermines for positioning of the lower screw in a more caudal position, which in turn decreases the stresses on the proximal femur [35]. In the present study, a periprosthetic femoral fracture occurred in one patient after a fall from standing position six months postoperatively. In this patient, the initial intertrochanteric fracture had healed and the patient was walking independently without an aid; therefore, this should not be regarded as an implant-related complication or a failure of the fracture healing process.

In conclusion, the dual head screw intramedullary hip nail is associated with high healing and low complication rates for patients with trochanteric fractures. The function of the patients is good or excellent in most cases; however, it declines, especially for those patients with age > 85 years, poor pre-fracture level of function, and AO/OTA-31-A3 fracture types.

## Conflict of interest

No conflicts of interest are declared by any author on this article.
